# Loss of PKM2 dysregulates inflammatory signaling in the infarcted murine heart

**DOI:** 10.14814/phy2.70193

**Published:** 2025-01-06

**Authors:** Katie C. Y. Lee, Allison L. Williams, Akitoshi Hara, Vedbar S. Khadka, Jeffrey Hayashi, Ralph V. Shohet

**Affiliations:** ^1^ Department of Medicine, John A. Burns School of Medicine University of Hawaii Mānoa Honolulu Hawaii USA; ^2^ Department of Cell and Molecular Biology, John A. Burns School of Medicine University of Hawaii Mānoa Honolulu Hawaii USA; ^3^ Bioinformatics Core, Department of Quantitative Health Sciences, John A. Burns School of Medicine University of Hawaii Mānoa Honolulu Hawaii USA; ^4^ John A. Burns School of Medicine University of Hawaii Mānoa Honolulu Hawaii USA

**Keywords:** fibrosis, inflammation, myocardial infarction, oxidative stress

## Abstract

Inflammation and a metabolic shift from oxidative metabolism to glycolysis are common in the ischemic heart, the latter partly controlled by pyruvate kinase (muscle, PKM). We previously identified alternative splicing promoting the PKM2 isoform after myocardial infarction (MI). We examined the role of PKM2 physiological upregulation after MI, modeled by ligation of the left anterior descending coronary artery, using global PKM2 knockout (PKM2^−/−^) mice. Echocardiography showed similar cardiac function between PKM2^−/−^ and control mice after MI. However, PKM2^−/−^ infarcted hearts had increased abundances of transcripts associated with oxidative stress and immune responses. Immunohistochemistry revealed greater abundance of macrophages in PKM2^−/−^ hearts prior to MI, with a small increase in CD86^+^ macrophages in PKM2^−/−^ infarcted hearts. Elevated baseline plasma IL‐6, IL‐1β, and C‐reactive protein, and cardiac IL‐6, 3 days post‐MI, were observed in PKM2^−/−^ mice. Oxidative lipid products were also elevated in baseline PKM2^−/−^ hearts, while antioxidant glutathione peroxidase 4 was reduced. Greater fibrosis was seen in PKM2^−/−^ hearts 28 days after MI. These findings suggest Pkm2 ablation primes the heart for increased oxidative stress, inflammation, and fibrosis post‐MI. The natural upregulation of PKM2 may mitigate fibrosis by reducing oxidative stress and inflammation, highlighting its protective role in the infarcted heart.

## 1 INTRODUCTION

During and after myocardial infarction (MI), there is a shift in cardiac energy production towards glycolysis (Greenwell et al., 2020; Lopaschuk & Stanley, 1997). The muscle form of pyruvate kinase (PKM) converts phosphoenolpyruvate to pyruvate in glycolysis, generating ATP. Our lab has shown differential expression of alternatively spliced PKM isoforms that was mediated by hypoxia‐inducible factor 1 (HIF‐1) after MI (Williams et al., 2018). PKM1 is the predominant isoform in healthy hearts and promotes oxidative metabolism. It decreases after MI with a concurrent increase in PKM2 expression. Hearts also have reduced pyruvate kinase activity after MI, despite the increase of PKM2. This decrease in enzymatic activity is likely due to the decrease in PKM1, in addition to dimerized PKM2 having lower activity compared to the tetrameric forms of PKM1 and 2 (Ibsen et al., 1981). This shift in PKM2 conformation is controlled by allosteric regulators and post‐translational modifications that are linked to upregulation of glycolysis, lactate production, and the pentose phosphate pathway (PPP) (Ibsen et al., 1981; Lv et al., 2011; Nagao et al., 1977). Increased PPP flux is particularly valuable during ischemia as it generates NADPH, a cofactor that provides hydrogen atoms for the reduction of ROS. Exogenous overexpression of PKM2 in cardiomyocytes has been shown to reduce oxidative damage (Magadum et al., 2020). We also recently described a role for PKM2 in the healthy heart as a buffer against excessive ROS associated with mitochondrial dysfunction, where deletion of PKM2 exacerbated superoxide production (Lee et al., 2024). The upregulation of PKM2 after MI may therefore have a similar function in the cardiac ischemic response.

Another important component of the initial cardiac response to ischemic injury is controlled by the immune system. ROS, among other factors, can react with cell components to form adducts that quickly attract neutrophils to the damaged myocardium, which then recruit macrophages to remove dead cells and later initiate wound healing (Dittrich & Lauridsen, 2019; Forte et al., 2018; Roh & Sohn, 2018). Neutrophils and macrophages rely on PKM2 to mount a proper pro‐inflammatory response (Doddapattar et al., 2022; Palsson‐McDermott Eva et al., 2015; Toller‐Kawahisa et al., 2023). Both PKM2 deletion and tetramerization limit the glycolytic capacity needed for activated macrophages, shifting them to an anti‐inflammatory phenotype (Doddapattar et al., 2022; Palsson‐McDermott Eva et al., 2015). While the effects of anti‐inflammatory cytokines are largely beneficial in reducing infarct size, a range of cytokines is needed for successful healing; an imbalance of specific pro‐ and anti‐inflammatory cytokines can be detrimental in cardiac remodeling (Ong et al., 2018).

Here, we provide evidence of a cardioprotective role of PKM2 against excessive inflammation. PKM2 ablation increased immune cell abundance and oxidative damage in the heart, with increased markers of systemic inflammation. The absence of PKM2 exacerbated fibrosis following MI. These data suggest that the dysregulated metabolism in PKM2^−/−^ hearts promotes oxidative stress that exacerbates the inflammatory response to infarction.

## 2 MATERIALS AND METHODS

### 2.1 Mice and reagents

C57BL6/J male and female mice between 8 and 12 weeks of age (>25 g) were used for experiments. All animal protocols and experiments were approved by the Institutional Animal Care and Use Committee of the University of Hawaii at Manoa (IACUC approval number 06–011‐17). All mice were housed in standard laboratory conditions with a 12 h light/dark cycle and provided food (5V5R, LabDiet) and water ad libitum. PKM2^fl/fl^ (stock no. 024048) and CMV‐Cre (stock no. 006054) mouse lines were acquired from the Jackson Laboratory. PKM2^−/−^ mice were generated as previously described (Lee et al., 2024). Briefly, PKM2^fl/fl^ mice contain loxP sites bracketing exon 10 of the *Pkm* gene, which is excised with Cre expression, leading to nonsense‐mediated decay of the transcript. After Cre recombination, PKM2^−/−^ mice were further bred to remove the Cre allele. Mice were euthanized by CO_2_; hearts were perfused with PBS and excised, frozen in liquid nitrogen, and stored at −80°C until further processing. Antibodies used in this study are listed in Table S1. Based on ejection fraction data, a sample size of 10 mice provided an 84% likelihood at *p* < 0.05 to see a 7.5% difference with a 4.7% standard deviation for inter‐animal variability.

### 2.2 Echocardiography

We used a 38 mHz transducer with a Vevo 2100 system (Fujifilm VisualSonics) to assess left ventricular function of unsedated mice with transthoracic echocardiography. M‐mode images were obtained in the left ventricular parasternal short‐axis view at the level of the papillary muscle. At least three consecutive heartbeats in M‐mode were used to measure fractional shortening (FS) and ejection fraction (EF) as previously described (Lee et al., 2024).

### 2.3 Myocardial infarctions

The left anterior descending artery (LAD) was permanently ligated to induce MI, as previously described (Brampton et al., 2014; Lee et al., 2024). Briefly, baseline echo was obtained, and mice were intubated and anesthesia maintained with isoflurane. A left‐lateral thoracotomy between the third and fourth rib and incision of the pericardial sac were performed to access the LAD for ligation with 7.0 silk. Myocardial infarction was confirmed by pallor and ST elevation, and the incision was closed in two layers. Mice were resuscitated on a warming pad until active. The ischemic free wall, including the infarct and border regions, was used for all tissue‐based assays. Six mice from each genotype died soon after LAD ligation and were excluded from analysis.

### 2.4 Western blotting

Protein extraction from frozen pulverized tissue of the left ventricle and isolated cardiomyocytes using 1X RIPA buffer, and western blotting and analyses were performed as previously described (Williams et al., 2018). Briefly, equal amounts of protein (30 μg) were loaded on 10% SDS‐PAGE (Tris–HCl) gels under reducing conditions and transferred to PVDF membranes. Intercept (PBS) Blocking Buffers (LI‐COR, cat.# 927–70,001) and Intercept T20 (PBS) Antibody Diluents (LI‐COR, cat.# 927–75,001) were used during blotting. Images were compiled for publication using Sciugo (Librach, 2023).

### 2.5 RNA isolation and semiquantitative real‐time PCR

Frozen left ventricles from 3 days post‐surgery were pulverized, and total RNA was obtained using the Qiagen RNeasy kit according to the manufacturer's instructions. The qScript cDNA synthesis kit (QuantaBio, cat.# 95,047–100) was used to reverse‐transcribe 1 μg of RNA for semiquantitative RT‐PCR (qPCR) with primers as described and the QuantiTect SYBR Green PCR Kit (Qiagen, cat.# 204,145). Primers were designed to span exon‐exon junctions when possible, and sequences are provided in Table S2. Samples were run on a QuantStudio 12 K Flex Real‐Time PCR System (Applied Biosystems). Tangerin (*Ehbp1l1*) was used for normalization as its expression has previously been shown to be unaffected by hypoxia (Bekeredjian et al., 2010). Relative abundance of transcripts was determined by ΔΔ*C*_t_ calculations according to standard methods.

### 2.6 RNA sequencing and analysis

Samples from male mice were used for RNA sequencing to minimize sex‐based transcriptomic variation (Yusifov et al., 2021). RNA was prepared as previously described (Williams et al., 2018). Total RNA was isolated as described above and quality checked using an Agilent 2100 Bioanalyzer. Only samples with an RNA integrity number ≥8 were used. Libraries were prepared and run on an Illumina NextSeq 500 system, and resulting reads were processed and analyzed as previously described (Lee et al., 2024). Infarction‐regulated transcripts were determined by comparison of sham and MI samples using a Benjamini‐Hochberg adjusted *p*‐value <0.01 and an absolute log_2_(fold change) ≥1.5 (corresponding to a fold change of ≥2.8 or ≤0.35). For comparisons between MI samples, transcripts with a Benjamini‐Hochberg adjusted *p*‐value <0.05 and an absolute log_2_(fold change) ≥1 (corresponding to a fold change of ≥2 or ≤0.5) were considered differentially expressed genes (DEGs). DEGs were then analyzed using the functional annotation tool in the Database for Annotation, Visualization, and Integrated Discovery (DAVID, v.6.8) to determine enriched gene ontology (GO) terms (Huang da et al., 2009; Sherman et al., 2022). Additionally, Ingenuity Pathway Analysis (IPA, Qiagen) was employed to assess gene expression patterns (Kramer et al., 2014). All primary RNA‐seq data are available on Gene Expression Omnibus under accession number GSE243668.

### 2.7 Immunohistochemistry (IHC)

Hearts fixed with 4% paraformaldehyde were embedded in paraffin and cut into 5 μm sections. Sections were deparaffinized, and antigen retrieval was performed with 10 mM sodium citrate buffer, pH 6. Images were obtained using the Leica THUNDER imaging system with deconvolution at 20X. Cross sections of cardiomyocytes were defined by an ovular shape surrounded by circular capillaries. For MI samples, cells in the border region near the ischemic zone were used for measurements. For sham samples, cells in the free wall were measured. Randomized fields were chosen along these regions. To maintain consistency, paraffin blocks were sectioned at the papillary muscle level, and cells from the subendothelial layer were used for measurements. At least 100 cardiomyocytes were measured per sample, and cross‐sectional areas were measured by ImageJ.

### 2.8 Picrosirius red staining

Hearts were fixed and embedded as described above. Sections were deparaffinized and rehydrated before staining with Weigert's Hematoxylin A and B (1:1, Electron Microscopy Sciences, cat. # 26044–05/26044–15). After washing, sections were incubated in picrosirius red solution (Direct Red 80 in saturated picric acid solution [Sigma Aldrich, cat. # 365548/P6744], 0.1% w/v%). Sections were then washed with acidified water and dehydrated in ethanol before clearing in xylene. Slides were then mounted with toluene mounting media (Richard‐Allan Scientific, cat. # 4112). Brightfield images were captured using the Leica THUNDER imaging system at 20X.

### 2.9 Hydroxyproline quantification

Paraffin‐embedded tissue sections were prepared and assayed for hydroxyproline using the QuickZyme Total Collagen Assay (QuickZyme Biosciences, cat. # QZBtotcol1) according to previously published methods (Baidoo et al., 2023). Briefly, heart tissues were sectioned and boiled in 6 M HCl at 95°C for 18 h. After centrifugation, supernatants were assayed for hydroxyproline. Assay results were normalized to tissue weights.

### 2.10 Multiplex assay

IL‐6 and IL‐1β abundance in citrated plasma and heart tissue were assessed using the Luminex® Discovery Assay Mouse Premixed Multi‐Analyte Kit (Bio‐Techne, cat. # LXSAMSM). Tissue samples were lysed in Cell Lysis Buffer (Thermo Fisher, cat. # FNN0011), diluted 1:5, and centrifuged. Equivalent amounts of total protein in tissue lysate and plasma samples were used. Assay was performed according to the manufacturer's protocol.

### 2.11 Cardiomyocyte isolation

Adult mouse cardiomyocytes were isolated using a Langendorff‐free method as previously described (Lee et al., 2024). Briefly, hearts were digested with collagenase until soft and pliable, at which point cells were dissociated from the tissue. Cardiomyocytes were purified by sedimentation while reintroduced to calcium. Approximately equivalent numbers of male and female mice were used for in vitro assays.

### 2.12 Enzyme‐linked immunosorbent assays

Blood was collected at the time of cardiac harvest in 0.5 M sodium citrate and spun to separate plasma. C‐reactive protein (CRP) levels in plasma were determined using a colorimetric assay (Invitrogen, cat. # EM20RB) according to manufacturer's instructions. 4‐hydroxynonenal (4‐HNE) was measured in isolated cardiomyocytes using according to the manufacturer's directions (Cell Biolabs, cat. # STA‐838).

### 2.13 Lipid profiling of cardiomyocytes

Following isolation, cardiomyocytes were transferred to extraction tubes with PBS. Lipids were extracted using a modified Bligh and Dyer extraction as previously described (Hsieh et al., 2021). Prior to biphasic extraction, a standard mixture of 75 lipid standards (Avanti 330,820, 861,809, 330,729, 330,727, 791,642) was added to each sample. Following two successive extractions, pooled organic layers were desiccated in a Thermo SpeedVac SPD300DDA. Lipid samples were resuspended in 1:1 methanol/dichloromethane with 10 mM ammonium acetate and transferred to robovials (Thermo 10,800,107) for analysis. Samples were analyzed by direct infusion on a Sciex 5500 with Differential Mobility Device (DMS) (comparable to Sciex Lipidyzer platform) with a targeted acquisition list consisting of 1450 lipid species across 17 subclasses according to previously published methods (Su et al., 2021). The DMS was tuned with EquiSPLASH LIPIDOMIX (Avanti 330,731). Data analysis was performed with in‐house data analysis workflow. Instrument settings, MRM lists, and analysis method are available (Su et al., 2021). Quantitative values were normalized to cell counts or plasma volume.

### 2.14 Statistics

Data was analyzed using GraphPad Prism 8. Unpaired student's *t*‐test to compare two groups and two‐way ANOVA with Tukey's test to compare means of three or more groups was used to test significance (*p* < 0.05). Error bars indicate standard deviation (SD). *p < 0.05, **p < 0.01, ***p < 0.001, ****p < 0.0001.

## 3 RESULTS

### 3.1 PKM2 deletion does not affect ejection fraction after MI

**FIGURE 1:**
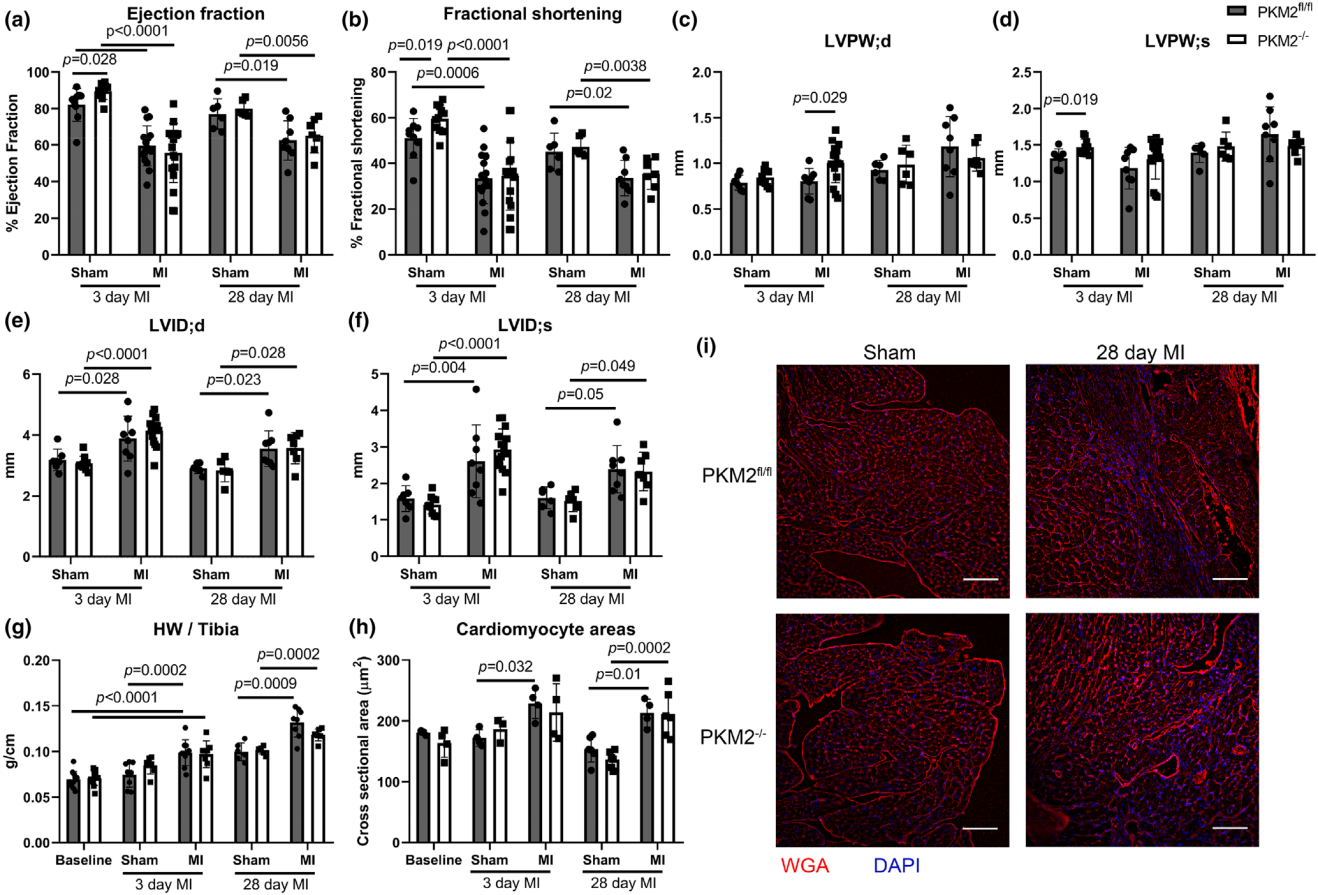
Heart function and hypertrophy after MI. (a) Ejection fraction, (b) fractional shortening, (c, d) left ventricular posterior wall thickness at diastole (d) and systole (s), and (e, f) left ventricular internal diameter of PKM2^fl/fl^ and PKM2^−/−^ hearts 3 days (*n* = 9–23 mice per group) or 28 days (*n* = 4–8 per group) following sham or MI surgery, (g) Heart weight to tibia length ratios measured at 3 (*n* = 7–12 per group) or 28 days (*n* = 4–8 per group) after surgery. (h) Quantification of cardiomyocyte cross‐sectional areas at baseline, or 3 days (*n* = 3–4 per group) or 28 days (*n* = 4–6 per group) after sham or MI surgery. (i) Representative images of cells delineated by wheat‐germ agglutinin (WGA), from sham and MI hearts taken 28 days post‐surgery. Scale bar = 100 μm. Data shown as means ± SD. Two‐way ANOVA with Tukey's multiple comparisons unless otherwise specified.

To elucidate the role of PKM2 in the ischemic response, we compared PKM2^−/−^ mice to controls (PKM2^fl/fl^) after myocardial infarction. Analysis of PKM isoform expression by western blotting demonstrated that control mice had reduced PKM1 and increased PKM2 protein expression, as we reported previously (Williams et al., 2018), whereas PKM2^−/−^ mice maintained elevated levels of PKM1 at baseline and after MI (Figure S1). Echocardiography showed that PKM2^−/−^ mice have preserved EF at baseline (Lee et al., 2024), with a decrement similar to PKM2^fl/fl^ mice at 3 or 28 days after MI (Figure 1a,b). Left ventricular posterior wall (LVPW) thickness and internal diameter (LVID) increased to showed similar increases between PKM2^fl/fl^ and PKM2^−/−^ mice (Figure 1c–f). Heart weight to tibia length ratios were similar in PKM2^fl/fl^ and PKM2^−/−^ mice after MI (Figure 1g). Similarly, cardiomyocyte cross‐sectional areas showed similar increases between PKM2^fl/fl^ and PKM2^−/−^ hearts at 3 and 28 days post‐MI (Figure 1h,i). These results suggest that the PKM2^−/−^ animals have grossly normal contractile and hypertrophic responses to MI.

### 3.2 Myeloid cells are more abundant in uninjured PKM2 ^−/−^ hearts

Previous studies have shown that PKM2 expression is upregulated after cardiac injury and can act as a co‐activator for transcription factors such as HIF‐1 and β‐catenin (Magadum et al., 2020; Palsson‐McDermott Eva et al., 2015). We therefore profiled the transcriptomes of PKM2^fl/fl^ and PKM2^−/−^ hearts 3 days after LAD ligation or sham surgery to assess the effects of PKM2 deletion in the early response to infarction. Principle component analysis and heatmap displayed distinct clustering of MI samples from baseline and sham samples, regardless of genotype (Figure S2A,B). However, additional analysis of MI samples alone revealed separate clusters for PKM2^fl/fl^ and PKM2^−/−^ hearts (Figure S2C), which indicated the loss of PKM2 altered the transcriptional response to injury.

**FIGURE 2:**
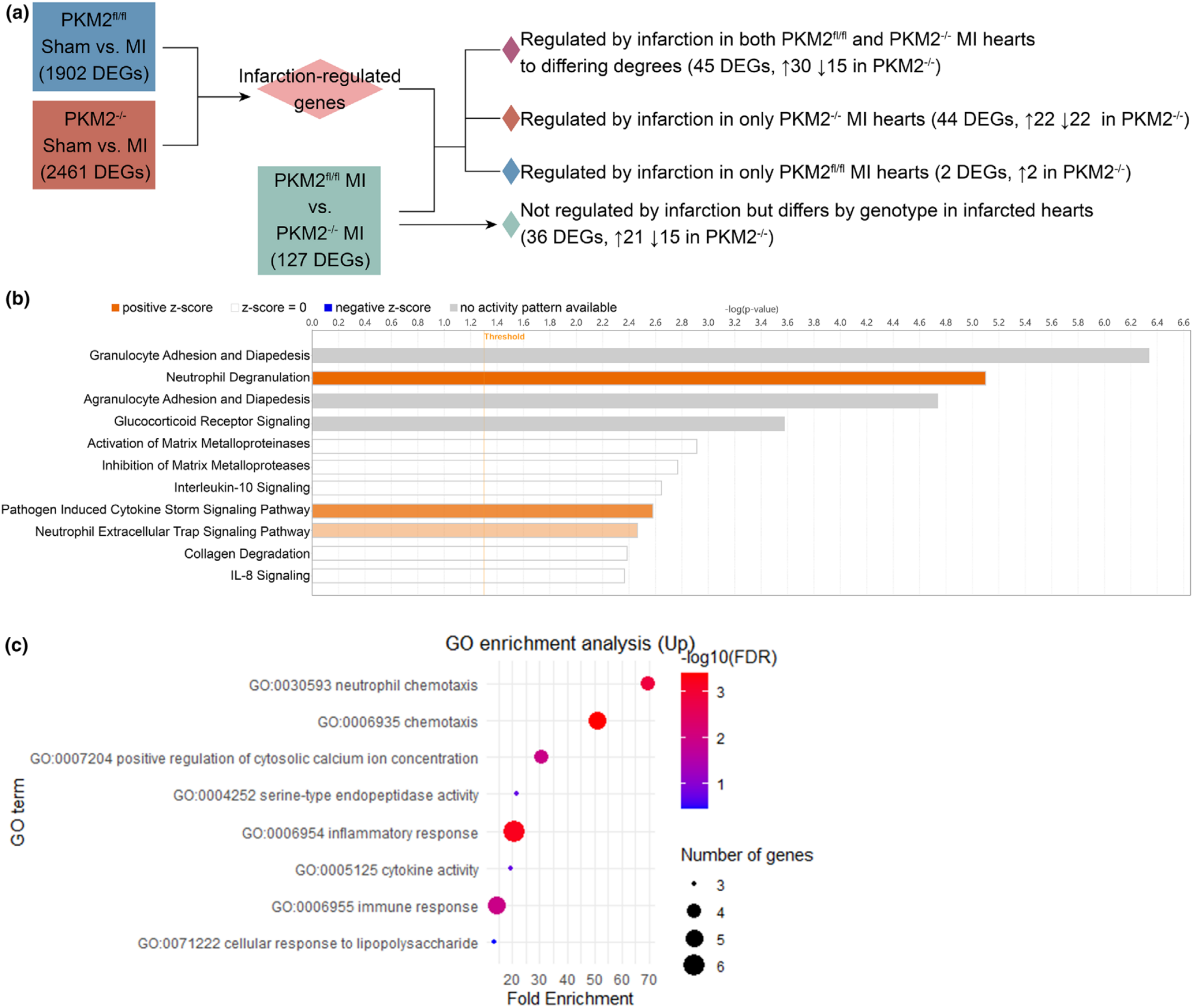
Differentially regulated genes for immune activity in PKM2^−/−^ hearts 3 days after MI. (a) Schematic of transcriptome analysis comparison method. Genes regulated by infarction were identified by comparing the transcriptomes of sham and MI samples. Subsequently, PKM2^fl/fl^ and PKM2^−/−^ hearts after MI were compared to identify genes dysregulated by the loss of PKM2. Up‐ and downregulated genes in PKM2^−/−^ hearts compared to PKM2^fl/fl^ hearts indicated by arrows in final analyses. (b) Enriched pathways by canonical pathway analysis of the 44 differentially expressed genes (DEGs) in PKM2^−/−^ infarcted hearts from Table S3, group 2. (c) Gene ontology (GO) term analysis of the DEGs with increased abundance in PKM2^−/−^ hearts after MI compared to PKM2^−/−^ MI hearts.

We first identified genes regulated by infarction by comparing sham and MI samples within their respective genotypes (Figure 2a). 1902 genes were differentially expressed in PKM2^fl/fl^ hearts after MI compared to PKM2^fl/fl^ shams, and 2461 differentially expressed genes (DEGs) were found in PKM2^−/−^ hearts after MI compared to PKM2^−/−^ shams. We considered these to be “infarction‐regulated genes”. We then performed a separate comparison with only PKM2^fl/fl^ and PKM2^−/−^ MI samples, yielding 127 DEGs. Of these, 91 DEGs were also infarction‐regulated genes, indicating both infarction and genotype were factors in their differential expression (Table S3). 44 DEGs were modified only in PKM2^−/−^ hearts after MI (Table S3, group 2, heatmap of representative genes shown in Figure S3).

Analysis of the 44 DEGs modified in only PKM2^−/−^ hearts after MI for enriched canonical pathways revealed enrichment of immune signaling pathways (Figure 2b). Gene ontology (GO) term analysis of the DEGs with increased abundance in PKM2^−/−^ hearts after MI attributed these genes to neutrophil activation and chemotaxis (Figure 2c). Abundance of key transcripts were validated by qPCR (Figure S4).

**FIGURE 3:**
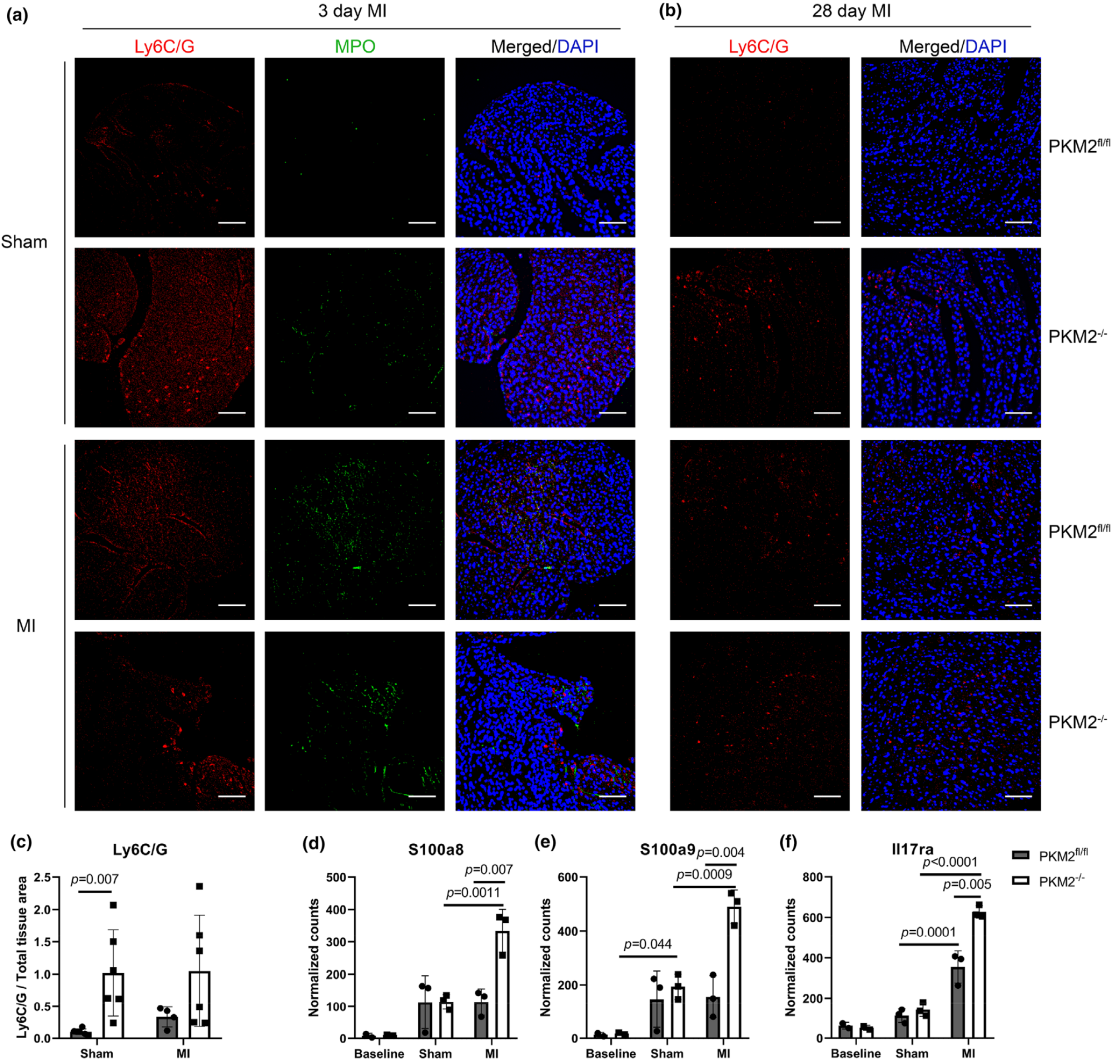
Elevated immune cell presence in PKM2^−/−^ hearts. (a, b) Representative images of myeloid and lymphoid staining (Ly6C/G, red) and myeloperoxidase (MPO, green) at the border region in PKM2^fl/fl^ and PKM2^−/−^ hearts 3‐ or 28‐days following sham or MI surgery. Scale bar = 100 μm. (c) Quantification of Ly6C/G staining in hearts 28 days after surgery. (d–f) Neutrophil transcript abundances (*S100a8*, *S100a9*, *Il17ra*) determined by RNA‐seq of PKM2^fl/fl^ and PKM2^−/−^ hearts at baseline or 3 days after sham or MI surgery (*n* = 2–3 per group). Data shown as means ± SD. Two‐way ANOVA with Tukey's multiple comparisons.

We therefore investigated immune cell populations in the heart. We probed for myeloid and lymphoid cells using an antibody against Ly6C/G, which showed abundant immune cells in both PKM2^fl/fl^ and PKM2^−/−^ hearts 3 days after MI (Figure 3a). Immune cell presence remained elevated in PKM2^−/−^ hearts 28 days after MI (Figure 3b,c). Surprisingly, Ly6C/G staining was also considerably higher in PKM2^−/−^ hearts after sham surgery alone compared to controls (Figure 3a–c). Because transcriptomic analysis suggested an increase of transcripts for genes involved in neutrophil activation and signaling in PKM2^−/−^ hearts after MI, such as *S100a8*, *S100a9*, and *Il17ra* (Figure 3d–f), we also co‐stained for myeloperoxidase which is predominantly expressed by neutrophils and has been shown to worsen tissue damage during inflammation and promote cardiovascular disease (Nicholls & Hazen, 2005). Myeloperoxidase staining was increased to a similar degree in both genotypes 3 days after MI but was more prominent in PKM2^−/−^ sham hearts compared to controls (Figure 3a).

**FIGURE 4:**
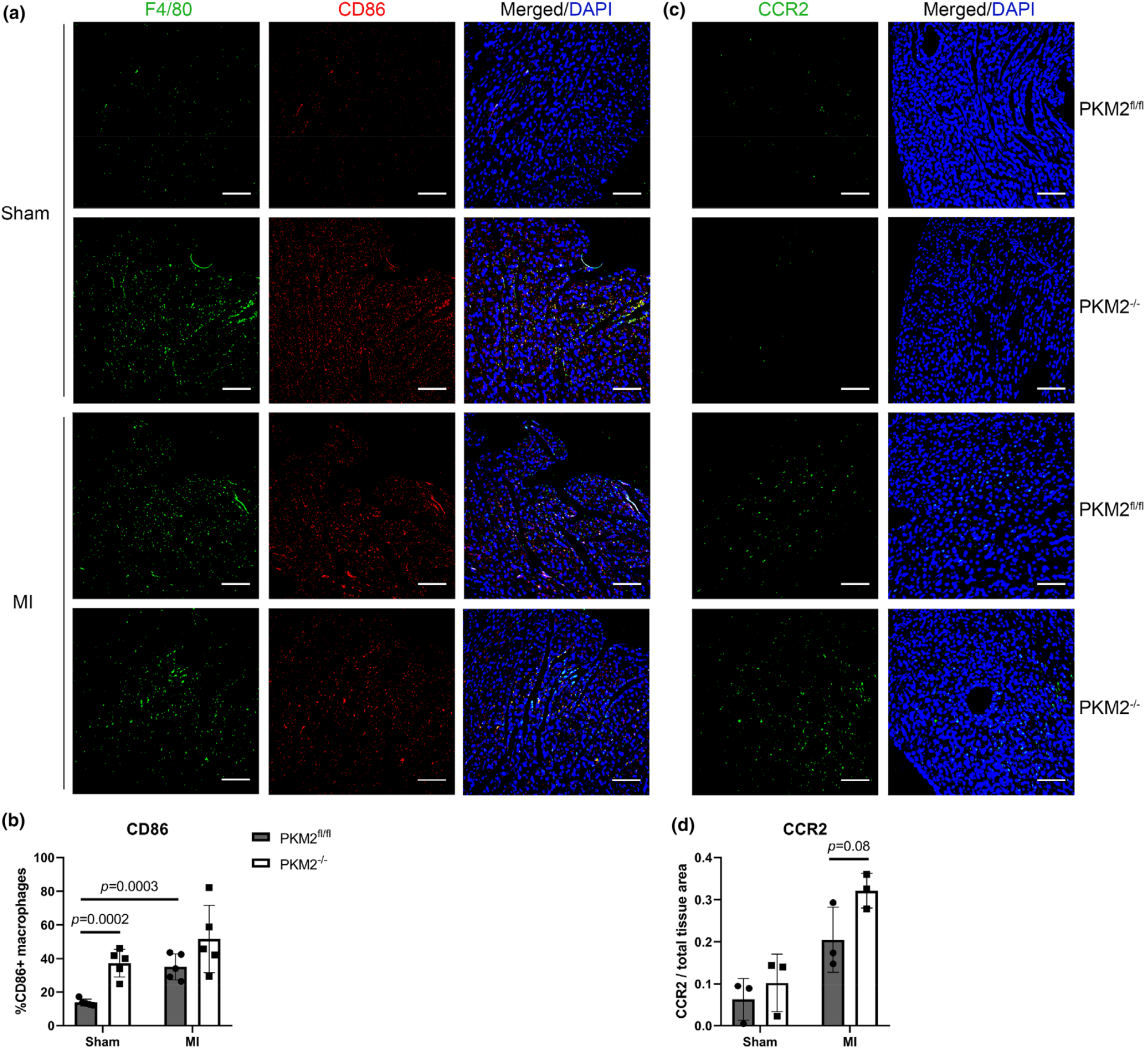
Increased macrophage presence in PKM2^−/−^ hearts. (a) Macrophages at the border region depicted by F4/80 (green), and pro‐inflammatory macrophages shown using the CD86 marker (red), in hearts 3 days after surgery. Scale bar = 100 μm. (b) Quantification of CD86^+^ macrophages in PKM2^fl/fl^ and PKM2^−/−^ hearts 3 days after sham or MI surgery (*n* = 5 hearts per group). (c) Resident and infiltrating macrophages differentiated by the CCR2 marker (green). Scale bar = 100 μm. (d) Quantification of CCR2 staining normalized to tissue area PKM2^fl/fl^ and PKM2^−/−^ hearts 3 days after sham or MI surgery (*n* = 3 hearts per group). Data shown as means ± SD. Two‐way ANOVA with Tukey's multiple comparisons.

Macrophages are a key source of matrix metalloproteinases, which were identified as enriched canonical pathways in PKM2^−/−^ MI hearts (Figure 2b) (Elkington et al., 2009). We characterized cardiac macrophages using MOMA‐2 which indicated comparable macrophage abundance between PKM2^fl/fl^ and PKM2^−/−^ 3 days after MI; however, like MPO staining, PKM2^−/−^ sham hearts had more MOMA‐2^+^ cells compared to sham controls (Figure S5A). Similar results were also observed using murine macrophage marker F4/80 (Figure 4a). We then probed additional markers to assess their activation status. CD86 is typically attributed to pro‐inflammatory macrophages, while CD206 is expressed on anti‐inflammatory and resident cardiac macrophages (Yunna et al., 2020). We observed increased abundance of CD86^+^ macrophages in both PKM2^−/−^ sham and MI hearts (Figure 4a,b), though this only reached statistical significance in sham animals. The abundance of CD206^+^ macrophages was not statistically different 3 days after surgery (Figure S5B,C). Although CCR2^−^ (C‐C chemokine receptor type 2) resident macrophages are usually present in the heart, most die during infarction and are replaced by CCR2^+^ macrophages derived from infiltrating monocytes (Sager et al., 2016; Yap et al., 2023). After detection of pro‐inflammatory macrophages in sham hearts, we used CCR2 staining to determine the origin of these macrophages. Interestingly, CCR2^+^ cells appeared to be more abundant in PKM2^−/−^ hearts after MI compared to PKM2^fl/fl^ controls, but this did not reach statistical significance (Figure 4c,d). Together, these results indicate that PKM2^−/−^ hearts have more myeloid cells in non‐infarcted sham mice, and these differences are less prominent after infarction.

### 3.3 Systemic inflammation is elevated in PKM2 ^−/−^ mice

**FIGURE 5:**
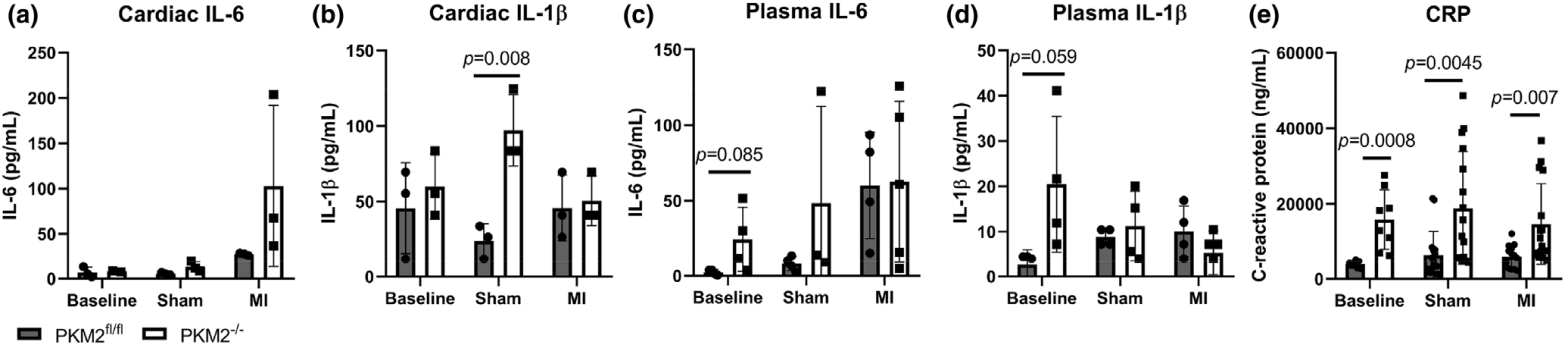
Loss of PKM2 increases systemic inflammation. (a, b) IL‐6 and IL‐1β levels in PKM2^fl/fl^ and PKM2^−/−^ hearts at baseline or 3 days after sham or MI surgery (*n* = 3 per group). (c, d) Circulating IL‐6 and IL‐1β levels in PKM2^fl/fl^ and PKM2^−/−^ plasma (*n* = 3–5 per group). (e) Circulating C‐reactive protein (CRP) in PKM2^fl/fl^ and PKM2^−/−^ plasma (*n* = 8–16 per group). Data shown as means ± SD. Two‐way ANOVA with Tukey's multiple comparisons.

The identification of pro‐inflammatory immune cells prompted an investigation into the cytokines released in the heart. Using a multiplex assay, we found an increase in IL‐6 in PKM2^−/−^ hearts compared to PKM2^fl/fl^ hearts 3 days after MI (Figure 5a). Surprisingly, IL‐1β, another pro‐inflammatory cytokine, was significantly higher in PKM2^−/−^ sham samples compared to PKM2^fl/fl^ controls, but this difference was not observed after MI (Figure 5b). We also measured IL‐6 and IL‐1β in the plasma and found elevated levels of both at baseline in PKM2^−/−^ mice compared to PKM2^fl/fl^ controls (Figure 5c,d). IL‐6 and IL‐1β also activate production of CRP, another inflammatory marker, in the liver (Ganter et al., 1989). We therefore probed for circulating CRP levels and found significantly elevated levels in PKM2^−/−^ mice compared to PKM2^fl/fl^ controls in all conditions (Figure 5e). These results indicated PKM2^−/−^ mice have systemic inflammation.

### 3.4 Oxidative damage and fibrosis are increased in PKM2 ^−/−^ hearts

**FIGURE 6:**
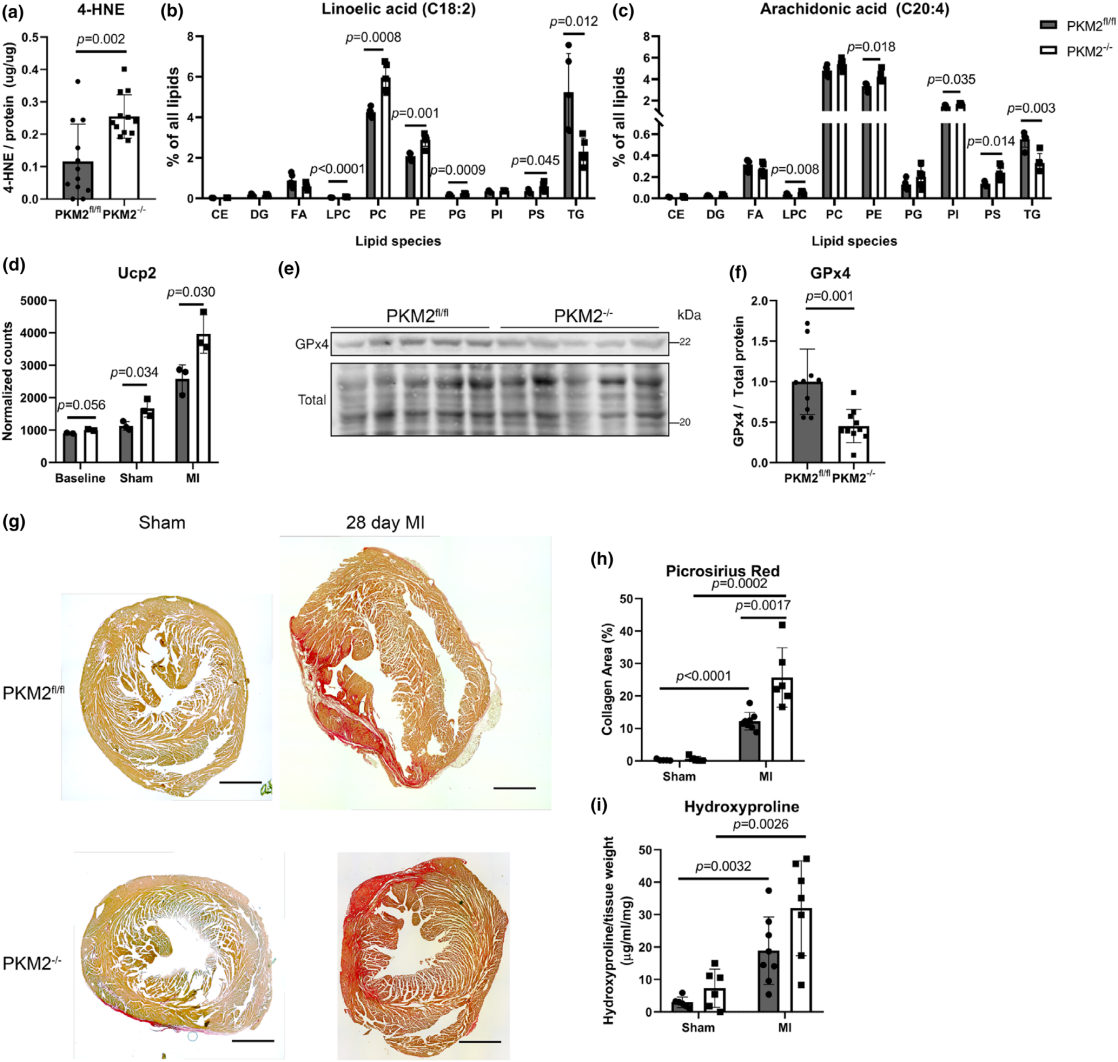
Oxidative stress and fibrosis are aggravated by loss of PKM2. (a) 4‐HNE measured in uninjured PKM2^fl/fl^ and PKM2^−/−^ hearts (*n* = 12 per group). Student's *t*‐test versus PKM2^fl/fl^ mice. (b, c) Linoleic and arachidonic acid abundance across lipid species in isolated cardiomyocytes. Student's *t*‐test versus PKM2^fl/fl^ mice. (d) *Ucp2* transcript abundance determined by RNA‐seq (*n* = 2–3 per group). (e, f) Representative western blot and quantification of GPx4 protein in left ventricle tissue (*n* = 10 per group). (g, h) Representative images and quantification of picrosirius red staining showing collagen area in hearts 28 days following sham or MI surgery (*n* = 5–8 per group). Scale bar = 1 mm. (i) Hydroxyproline quantified in hearts 28 days after MI (*n* = 5–8 per group). Data shown as means ± SD. Two‐way ANOVA with Tukey's multiple comparisons unless otherwise specified.

To better understand the underlying causes for increased myeloid cells and inflammatory markers in uninjured PKM2^−/−^ hearts, we examined products of oxidative stress, which are known to trigger inflammation. We found substantially elevated levels of 4‐hydroxy 2‐nonenal (4‐HNE), the major lipid peroxidation product (Dalleau et al., 2013), in PKM2^−/−^ cardiomyocytes compared to controls at baseline (Figure 6a). In support of this, we profiled the lipids in healthy PKM2^fl/fl^ and PKM2^−/−^ cardiomyocytes and identified an increase of 4‐HNE precursors (linoleic acid and arachidonic acid) in membrane lipids (Figure 6b,c) (Dalleau et al., 2013). There is evidence that 4‐HNE adducts can form in mitochondria under conditions of elevated ROS and promote dysfunction (Santin et al., 2019). We previously reported elevated levels of mitochondrial superoxide in cardiomyocytes, with a substantial increase in total ROS after hypoxic (1% O_2_) treatment in PKM2^−/−^ cardiac myocytes (Lee et al., 2024). Mitochondrial uncoupling proteins 2 and 3 (UCP2/3) are also acutely activated by ROS (Mailloux & Harper, 2011). Specifically, UCP2 regulates the polarization of the inner mitochondrial membrane to limit ROS production, and transcriptomic analysis revealed increased abundance of *Ucp2* in infarcted PKM2^−/−^ hearts compared to controls (Figure 6d, Figure S3C).

Formation of 4‐HNE is typically controlled by glutathione peroxidase 4 (GPx4), a key antioxidant enzyme (H‐f et al., 2021). Interestingly, GPx4 protein is also reduced in PKM2^−/−^ hearts at baseline (Figure 6e,f). Cardiac remodeling after infarction can be impaired by elevated ROS levels through activation of immune cells in response to peroxidation products (Dhalla et al., 2022; Wójcik et al., 2021). We therefore stained for collagen deposition in hearts 28 days after sham or MI using picrosirius red to assess fibrosis. We found a larger fibrotic area in PKM2^−/−^ hearts compared to controls 28 days after MI that was reflected in hydroxyproline concentrations (Figure 6g–i). These results demonstrate that loss of PKM2 leads to increased oxidative stress before injury, which translates to greater fibrosis after infarction.

## 4 DISCUSSION

PKM2 is conventionally recognized as an important enzyme for glycolysis and energy production, but we have also recently demonstrated its role in mitigating oxidative stress. Here, we have shown that when PKM2 is absent, the increase in oxidative damage can exacerbate inflammatory signaling and impair tissue repair after infarction.

Our findings suggest that complete deletion of PKM2 in mice leads to chronic pro‐inflammatory signaling, exacerbated inflammation, and sustained CD86^+^ macrophage activation after infarction, which correlates with worse patient outcomes (O'Rourke et al., 2019). It is possible that immune cells in PKM2^−/−^ mice are primed for pro‐inflammatory signaling that perpetuates a vicious cycle of local and systemic inflammation. Future studies will be needed to characterize the sensitivity of macrophage activation more extensively. Imbalance of differentially polarized macrophages can have damaging consequences after infarction. Heightened pro‐inflammatory macrophage activity exacerbates cell death and limits tissue repair (Duncan et al., 2020). Consonant with this, we observed a greater fibrotic area in PKM2^−/−^ infarcted hearts.

CRP is a prominent biomarker for inflammation and cardiovascular risk and was recently found to be a marker for metabolic syndrome (Devaraj et al., 2009). CRP functions in pro‐inflammatory signaling and wound healing appear to depend on concentration, conformation, and activating factors (Del Giudice & Gangestad, 2018). It is an established activator of monocytes and facilitates their recruitment to the site of inflammation (Han et al., 2004; Sproston & Ashworth, 2018). Elevated CRP correlated with larger infarct size in patients with MI, suggesting an initial pro‐inflammatory role (Orn et al., 2009). The high levels of CRP present in PKM2^−/−^ mice regardless of cardiac injury suggest PKM2 deficiency induces inflammation and cardiac risk. Several studies have also demonstrated increased superoxide production associated with elevated CRP levels (Devaraj et al., 2009). We have previously shown increased abundance of mitochondrial superoxides in PKM2^−/−^ cardiomyocytes compared to controls, as well as impaired insulin‐induced glucose uptake and accumulation of intracellular lipids (Lee et al., 2024). These data illustrate a heightened inflammatory state in PKM2^−/−^ hearts that may increase underlying risk for cardiovascular disease.

CRP is produced in the liver in response to inflammatory signals that trigger increased secretion of pro‐inflammatory cytokines IL‐6 and IL‐1β (Krayem et al., 2017; Rhodes et al., 2011). We found both circulating IL‐6 and IL‐1β were increased in PKM2^−/−^ mice at baseline. Our results are substantiated by a previous study that found increased serum IL‐6 in aged PKM2^−/−^ mice (Dayton et al., 2016). Although activated macrophages are the major producers of IL‐6 and IL‐1β, a wide array of immune cells and stromal cells can also produce these cytokines (Gubernatorova et al., 2018; Kaneko et al., 2019).

In the list of 44 DEGs that were regulated by infarction only in PKM2^−/−^ hearts were *Adrb1*, *Grb14*, and *Kbtbd12*. These genes are predominantly expressed in cardiac or muscle cells compared to immune cells and therefore are of interest for future study as part of a cardiac‐specific response to PKM2 ablation. We observed decreased abundance of these transcripts in PKM2^−/−^ infarcted hearts (Table S3). There is some evidence of Adrb1 as an anti‐inflammatory mediator (Evans et al., 2020). Lower abundance of *Adrb1* transcript is also commonly found in patients with heart failure (Li et al., 2005). Interestingly, loss of Grb14 can induce cardiomyopathy with increased fibrosis (Lin et al., 2010). It is possible that reduction of these RNAs in PKM2^−/−^ hearts contributed to the enhanced fibrotic response to infarction.

We observed an elevated abundance of transcripts for myeloid chemoattractants in PKM2^−/−^ infarcted hearts, including C‐X‐C motif chemokine ligands (CXCL) 1, 3, 5, and C‐X‐C motif chemokine receptor 2 (CXCR2), that may support myeloid cell infiltration in PKM2^−/−^ hearts. Infiltrating neutrophils are an important part of the immune response to infarction, but typically clear within a day (Forte et al., 2018). Myeloperoxidase is most commonly associated with neutrophils, but can also be expressed by macrophages and monocytes (Gurski & Dittel, 2022). Our results suggest that immune cell populations are elevated in PKM2^−/−^ hearts and sustained to 28 days after infarction. Prolonged pro‐inflammatory activity of immune cells can damage the surrounding tissue by excessive extracellular matrix degradation and oxidative stress (Carbone et al., 2013).

The oxidative burst from neutrophils can also increase lipid peroxidation products, and 4‐HNE can elevate pro‐inflammatory signaling via NF‐κB (Wójcik et al., 2021). Our observation of increased 4‐HNE in baseline PKM2^−/−^ cardiomyocytes indicate high oxidative stress that promotes activation of immune cells and contributes to impaired cardiac remodeling following infarction. Indeed, enhanced cardiac remodeling has been demonstrated in infarcted and ischemia/reperfused hearts with increased 4‐HNE abundance and reversed by limiting 4‐HNE production (Santin et al., 2019; Zhai et al., 2021). The increase in *Ucp2* transcripts we observe may be a response to elevated ROS generation that we demonstrated previously in hypoxic PKM2^−/−^ cardiomyocytes (Lee et al., 2024). In sum, these results suggest the elevated inflammatory state in the PKM2^−/−^ heart contributes to oxidative stress that damages the myocardium.

The present study suggests that PKM2^−/−^ cardiac cells may be primed for an amplified stress response due to heightened inflammation. Our data suggest that the higher basal inflammation and oxidative states may predispose and contribute to the greater fibrotic area observed in PKM2^−/−^ hearts after MI. It is possible that these factors initially contribute to a larger infarct size, which will need to be assessed in future studies. While fibrosis is beneficial in maintaining the structural integrity of the heart, stiffness can impair ventricular function that eventually leads to heart failure (Kong et al., 2014). However, a larger area of collagen deposition could also be a result of greater necrosis due to a larger infarction. Our previous results showed reduced cell viability of PKM2^−/−^ cardiomyocytes in hypoxia (Lee et al., 2024) which might suggest that infarct size could be larger in PKM2^−/−^ hearts (although we do not see greater ventricular dysfunction that would be anticipated from a larger infarct). Future studies will be needed to distinguish the injury and wound healing responses. Earlier assessments of the infarct size in acute MI may shed light on the cause of increased collagen deposition in PKM2^−/−^ infarcted hearts. Another concern is that variability in infarct size among assessed samples could contribute to the observed variance in leukocyte numbers, though similar variability in sham samples suggests it may stem from inter‐animal differences. Despite this, we still observe a greater abundance of leukocytes in PKM2^−/−^ sham hearts compared to control shams that is statistically significant.

The underlying inflammation in the PKM2 knockout adds complexity in assessing the response to infarction. The PKM2 dimer supports the glycolytic burst needed in activated immune cells, whereas tetramerization using small molecule activators such as TEPP‐46 reduces the pro‐inflammatory and phagocytic capabilities of T‐cells, macrophages, natural killer cells, and dendritic cells (Angiari et al., 2020; Jin et al., 2020; Palsson‐McDermott Eva et al., 2015; Walls et al., 2020; Yi et al., 2020). The response to PKM2 deletion or knockdown seems to be specific to each cell type, from incomplete activation of T‐cells to shifting macrophages towards the anti‐inflammatory M2 phenotype (Damasceno et al., 2020; Doddapattar et al., 2022). These may lead to less efficient removal of debris and dead cells, ultimately exacerbating inflammation. In our study, we observed elevated inflammatory markers, oxidative damage, and fibrosis in PKM2^−/−^ mice following MI. It may be that the chronic inflammation has shifted these immune cells to a pro‐inflammatory state, which is then aggravated upon ischemic injury. The global deletion of PKM2 also presents some confounding effects, making it challenging to distinguish the specific role of PKM2 in cardiomyocytes from its role in myeloid cells. As demonstrated in angiotensin‐II‐treated neonatal rat cardiac fibroblasts and myocytes, PKM2 may play a pro‐fibrotic role by promoting oxidative stress (Zhang et al., 2021). Our results showing increased oxidative damage in PKM2^−/−^ hearts supports the idea that PKM2 expression needs to be fine‐tuned to prevent oxidative stress and excessive cardiac remodeling.

In summary, our study provides evidence that global loss of PKM2 leads to chronic inflammation and larger cardiac myeloid cell populations with increased ROS generation. This underlying oxidative damage leaves heart tissue more susceptible to ischemic injury and fibrosis. These findings highlight the importance of maintaining the delicate balance between oxidative stress and metabolism to preserve cardiac function.

## AUTHOR CONTRIBUTIONS

RVS, ALW, and KCYL conceived and designed research. KCYL, AH, and JH performed experiments, KCYL, VSK, and JH analyzed results, and KCYL, ALW, RVS interpreted results of experiments. KCYL drafted the manuscript and prepared figures, RVS, ALW, KCYL revised and edited the manuscript. KCYL, ALW, AH, VSK, JH, and RVS approved the final version of the manuscript.

## FUNDING INFORMATION

This work was supported by the National Institutes of Health [grant numbers P30 GM103341 to RVS, T32 HL115505 to ALW and KCYL, P30 CA‐071789 and 5G12 MD‐007601 to the Microscopy and Imaging Core, P20 GM‐103466, U54 GM138062, and 5P30 GM‐114737 to the Bioinformatics Core, U54 MD007601 to the Histology Core Facility, Shared Instrumentation grant 1S10OD028515–01 to the MIFCC, and P20GM103451 to the NM‐INBRE]; and the American Heart Association [grant number 23PRE1027246 to KCYL].

## CONFLICT OF INTEREST STATEMENT

None declared.

## ETHICS STATEMENT

All animal protocols and experiments were approved by the Institutional Animal Care and Use Committee of the University of Hawaii at Manoa (IACUC approval number 06–011‐17).

## Supporting information


Figure S1.

Table S1.

## Data Availability

RNA‐seq data are available in the National Center for Biotechnology Information Gene Expression Omnibus Repository (series GSE243668). Data are also available from the corresponding author upon reasonable request.
