# Supplementary material for: Loss of PKM2 dysregulates inflammatory signaling in the infarcted murine heart
Source: Physiol Rep. 2025 Jan 6;13(1):e70193. doi: 10.14814/phy2.70193 (PMC11705480; doi:10.14814/phy2.70193)
Supplement: Supplementary file 1 — Figure S1. [file PHY2-13-e70193-s002.docx]

FIGURE S1


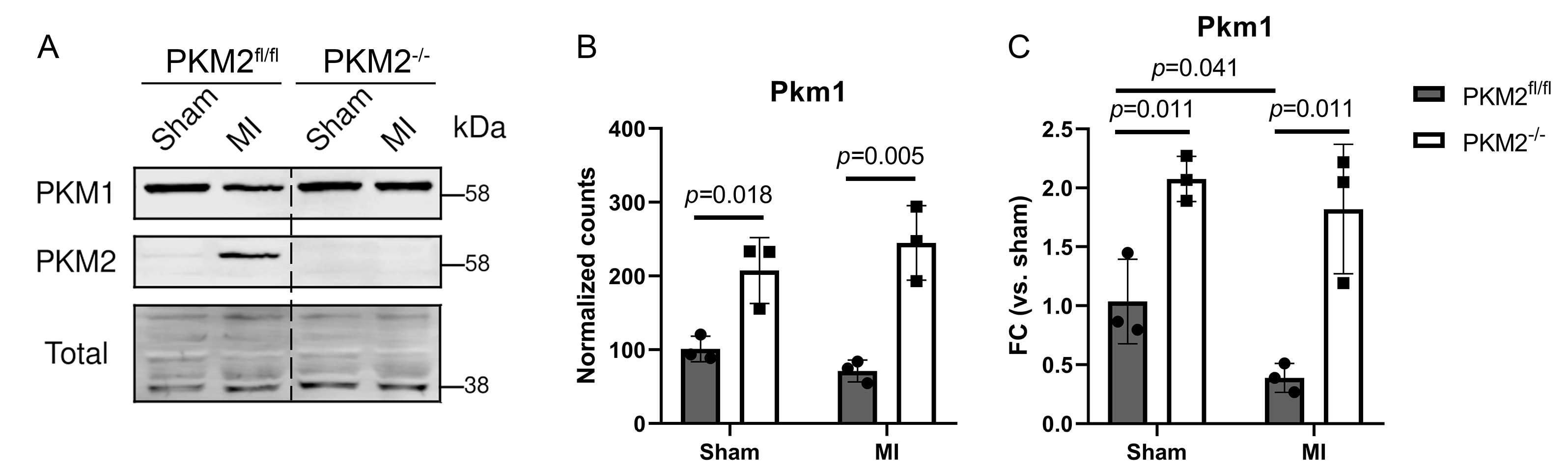


Figure S1. Increased PKM1 abundance in PKM2^-/-^ mice is sustained after MI.

A. Western blot of PKM1 and PKM2 in PKM2^fl/fl^ and PKM2^-/-^ hearts, 3 days following sham or MI surgery. Dotted line delineates connection of non-consecutive lanes on the same membrane. B-C. *Pkm1* transcripts 3 days following sham or MI surgery, assessed by RNA-sequencing or semiquantitative PCR (qPCR), respectively (n=2-3 hearts per group). Two-way ANOVA with Tukey’s multiple comparisons. Data shown as means ± SD.

FIGURE S2


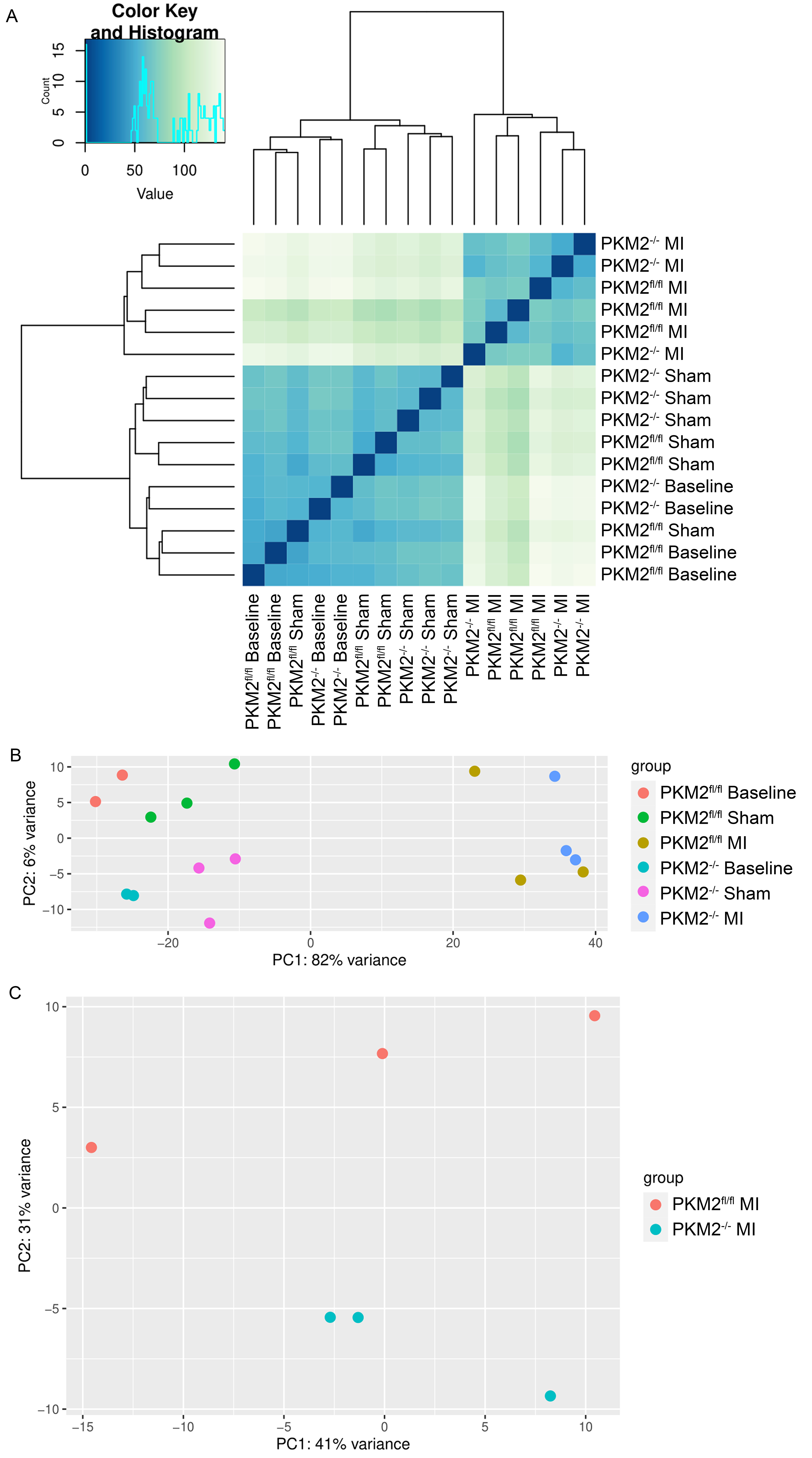


Figure S2. Clustering of PKM2^fl/fl^ and PMK2^-/-^ transcriptomes.

A. Heatmap and dendrogram showing similarity of PKM2^fl/fl^ and PKM2^-/-^ cardiac transcriptomes at baseline or 3 days after sham or MI surgery. B. Clustering of transcriptomes visualized by principle component analysis (PCA) plots. C. PCA plot showing distinct clustering of PKM2^fl/fl^ and PKM2^-/-^ MI samples.

FIGURE S3


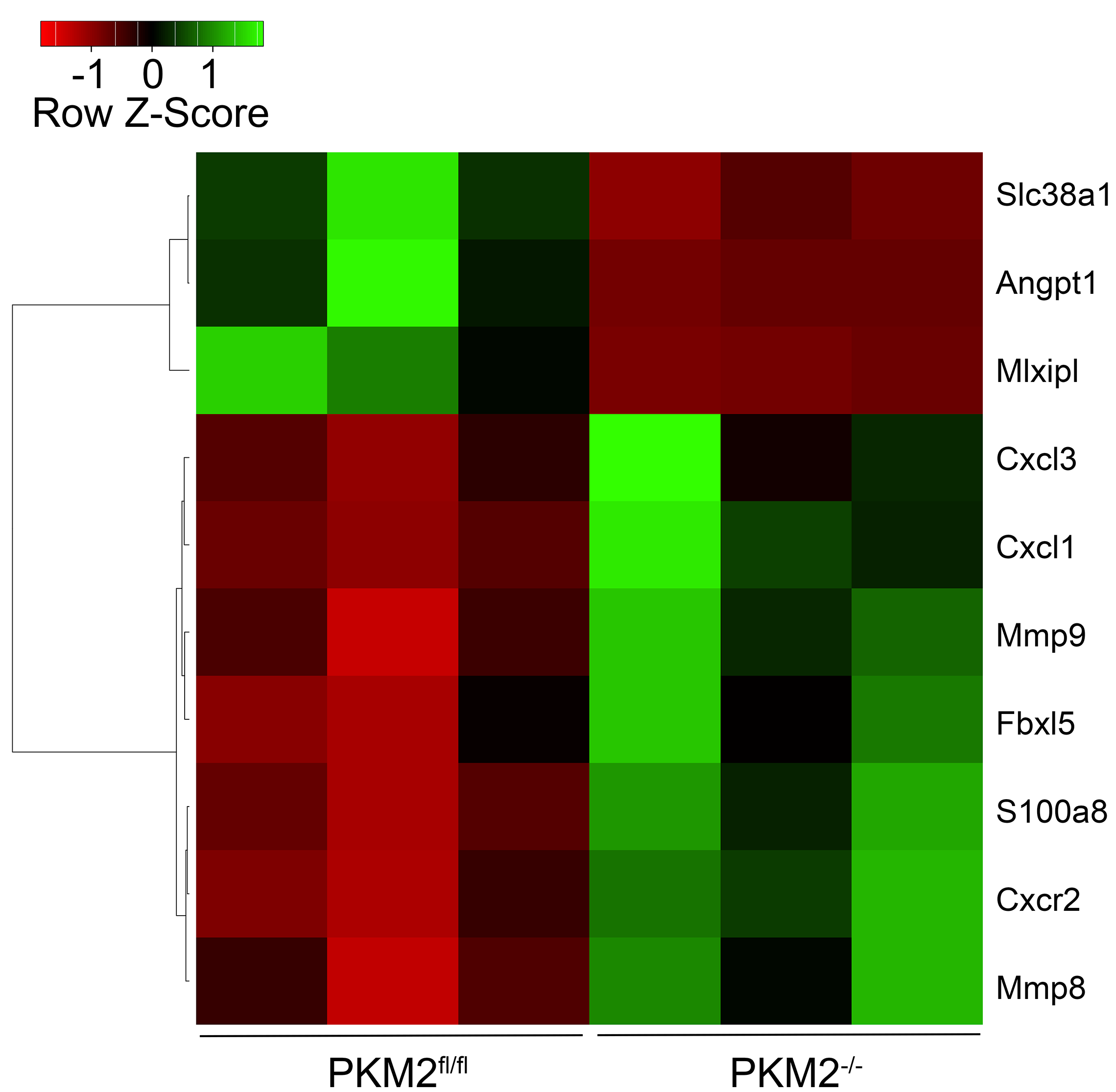


Figure S3. Representative DEGs dysregulated in PKM2^-/-^ infarcted hearts.

Representative genes of the 44 DEGs identified to be only dysregulated in PKM2^-/-^ infarcted hearts by RNA-seq analysis. Genes clustered by average linkage and Pearson’s correlation to calculate distances.

FIGURE S4


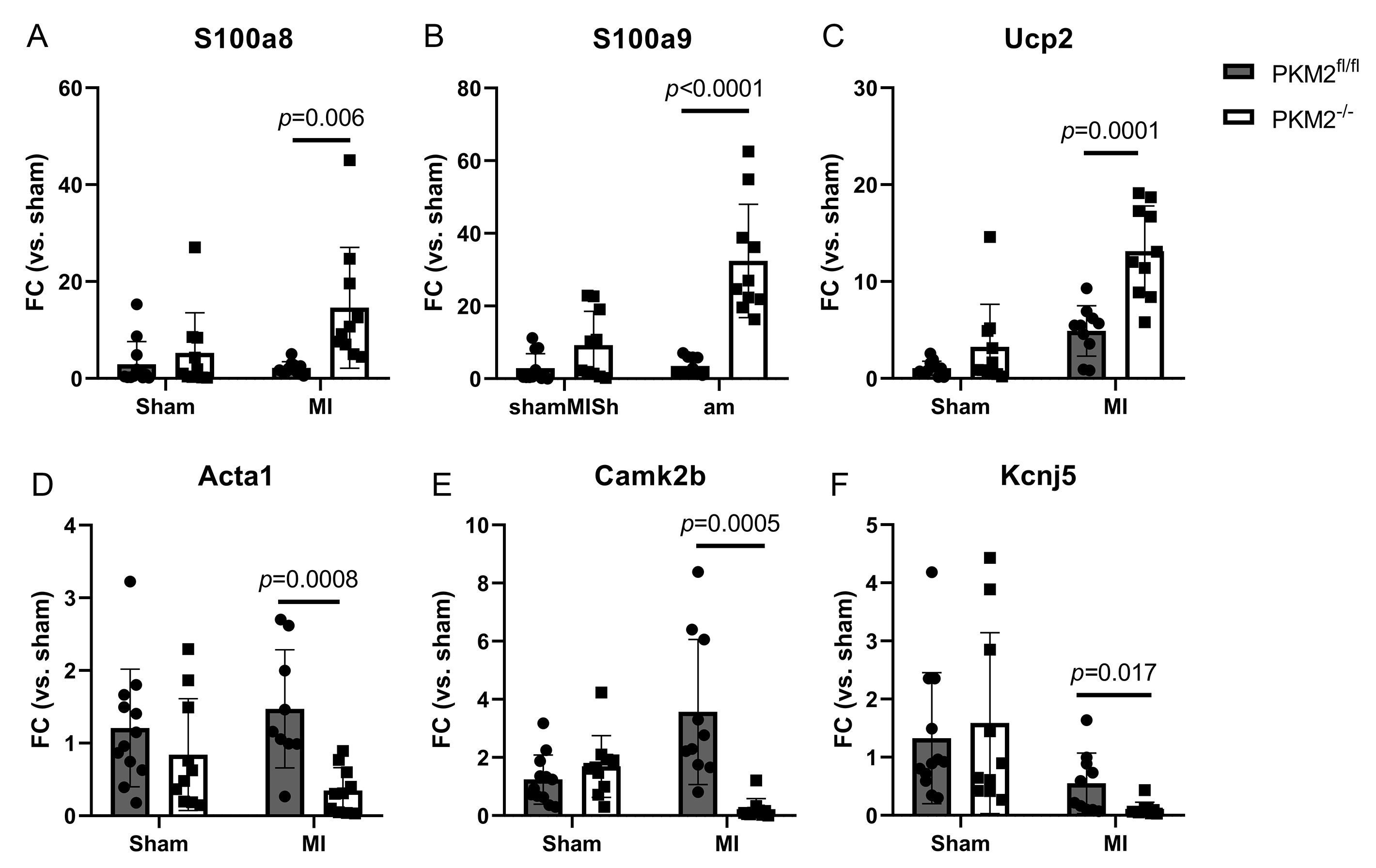


Figure S4. Validation of RNA-seq DEGs by qPCR.

A-B. Transcripts of genes relating to neutrophils, C. oxidative stress, D-F. and cardiac contraction were assessed by qPCR of left ventricle tissue (n=10 per group). Two-way ANOVA with Tukey’s multiple comparisons. Data shown as means ± SD.

FIGURE S5


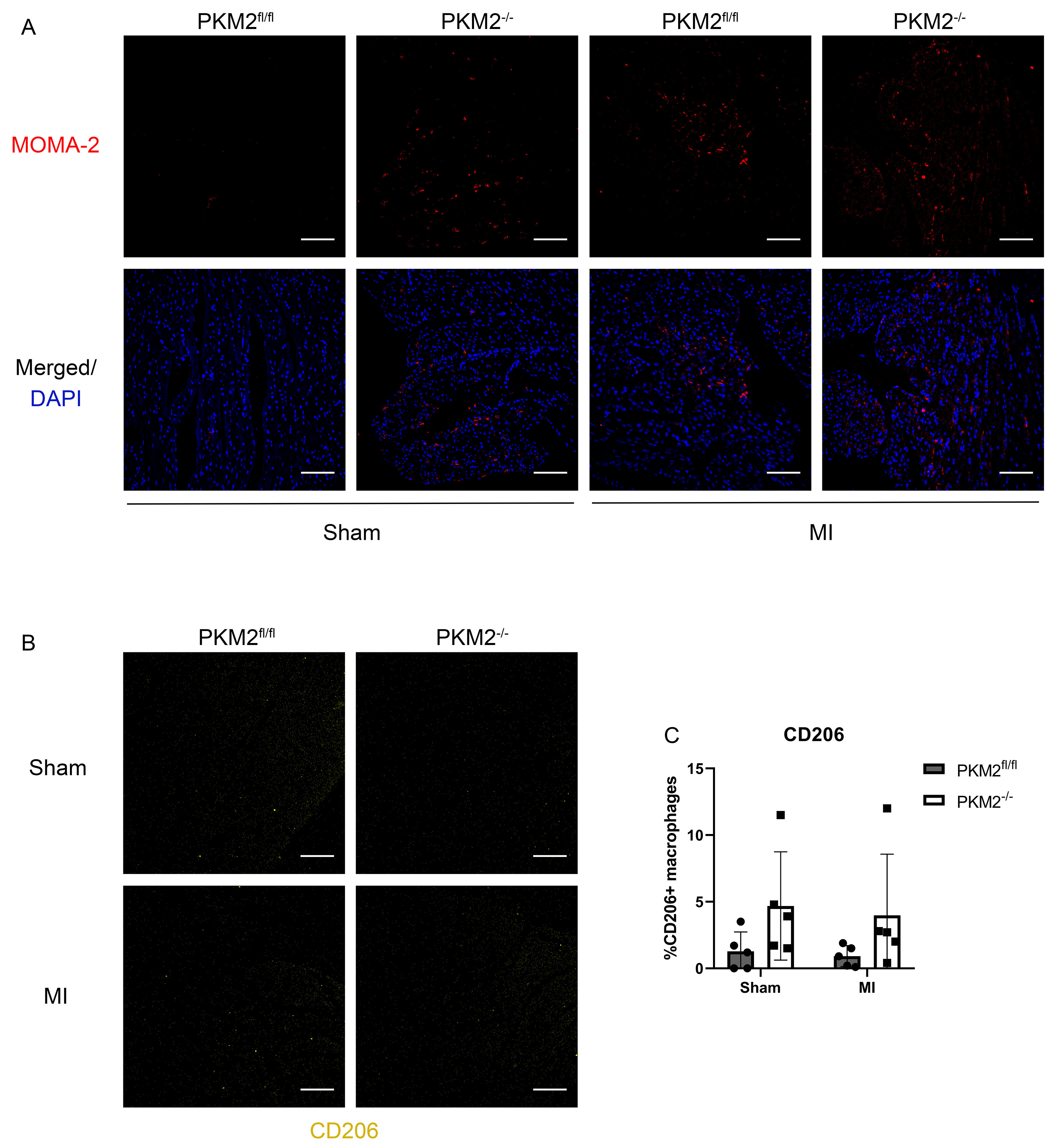


Figure S5. Increased abundance of macrophages in PKM2^-/-^ hearts.

A. Macrophages depicted by MOMA-2 (red), a murine macrophage antibody, in PKM2^fl/fl^ and PKM2^-/-^ hearts 3 days after sham or MI surgery. Scale bar=100μm. B. Anti-inflammatory macrophages identified by the marker CD206 in PKM2^fl/fl^ and PKM2^-/-^ hearts 3 days after sham or MI surgery. Scale bar=100μm. Stained sections are also shown in Figure 4 for F4/80 and CD86 staining. C. Quantification of CD206^+^ macrophages. Two-way ANOVA with Tukey’s multiple comparisons. Data shown as means ± SD.
