# Supplementary material for: Loss of PKM2 dysregulates inflammatory signaling in the infarcted murine heart
Source: Physiol Rep. 2025 Jan 6;13(1):e70193. doi: 10.14814/phy2.70193 (PMC11705480; doi:10.14814/phy2.70193)
Supplement: Supplementary file 2 — Table S1. [file PHY2-13-e70193-s001.docx]

| **Table S1: Antibodies used in this study** | | | | |
| --- | --- | --- | --- | --- |
| **Antigen** | **Dilution** | **Company** | **Catalog number** | **RRID** |
| PKM1 | WB 1:4000 | Proteintech | 15821-1-AP | AB_2163820 |
| PKM2 | WB 1:4000 | Proteintech | 15822-1-AP | AB_1851537 |
| GPX4 | WB 1:1000 | Abcam | ab185689 | AB_3271574 |
| IRDye 800CW Donkey anti-Rabbit IgG | WB 1:20000 | LI-COR | 926-32213 | AB_621848 |
| IRDye 680RD Donkey anti-Mouse IgG | WB 1:20000 | LI-COR | 926-68072 | AB_10953628 |

Table S1. Antibodies used in this study.

Primary and secondary antibodies used for western blotting listed with Research Resource Identifiers (RRID).

| **Table S2: qPCR primer sequences** | | | |
| --- | --- | --- | --- |
| Gene | Forward | Reverse | Length (bp) |
| Pkm1 | CACCGTCTGCTGTTTGAAGA | AGCACTCCTGCCAGACT | 144 |
| Pkm2 | CATCTACCACTTGCAGCTATTC | GAGCACTCCTGCCAGACT | 152 |
| Ehbp1l1 | TTCCAGTTTGTGGCGTGTTAC | TTCCGCCGAGTCCATACCA | 91 |
| Ucp2 | TCCCCTGTTGATGTGGTCAA | CAGTGACCTGCGCTGTGGTA | 70 |
| Camk2b | CCGTTACTCCTGAAGCCAAA | CTTCAGACATTCCACAGTCTCC | 158 |
| S100a8 | CAAGGAAATCACCATGCCCTCTA | ACCATCGCAAGGAACTCCTCGA | 158 |
| S100a9 | TGGTGGAAGCACAGTTGGCAAC | CAGCATCATACACTCCTCAAAGC | 128 |
| Kcnj5 | CTTGGAAGGATCTCCAGAAGTTAG | GTGCTGTTGGAGGATGAAGT | 177 |
| Acta1 | GCTCTTCCAGCCTTCCTTTATC | TCAGCGATACCAGGGTACAT | 153 |

Table S2. qPCR primer sequences.

Primers used for qPCR, designed to span exon-exon junctions when possible.

| **Table S3: RNA-seq DEGs** | | | | | | | | | | | | | | | | | | | |
| --- | --- | --- | --- | --- | --- | --- | --- | --- | --- | --- | --- | --- | --- | --- | --- | --- | --- | --- | --- |
|  | | | | | | PKM2^fl/fl^ | | | | | | | PKM2^-/-^ | | | | | | |
| Group | Gene name | log_2_Fold  Change | pvalue | padj | Sham | | | | MI | | | Sham | | | | MI | | |  |
| 1 | Abca12 | -1.54 | 6.28E-04 | 3.36E-02 | 297 | | 212 | 152 | 56 | 58 | 30 | 108 | | 163 | 110 | 16 | 16 | 17 |  |
| 1 | Abca13 | 1.28 | 7.80E-04 | 3.79E-02 | 8 | | 8 | 4 | 23 | 23 | 42 | 11 | | 18 | 2 | 81 | 71 | 65 |  |
| 1 | Aqp7 | -1.61 | 1.45E-06 | 4.17E-04 | 618 | | 805 | 729 | 261 | 361 | 131 | 640 | | 462 | 559 | 85 | 86 | 75 |  |
| 1 | Card11 | -1.23 | 6.93E-05 | 7.86E-03 | 24 | | 29 | 30 | 204 | 350 | 242 | 53 | | 34 | 23 | 72 | 146 | 124 |  |
| 1 | Cd300lf | 1.14 | 1.19E-06 | 3.61E-04 | 60 | | 69 | 20 | 649 | 392 | 694 | 68 | | 90 | 59 | 1578 | 1156 | 1112 |  |
| 1 | Clec4e | 1.36 | 6.19E-06 | 1.38E-03 | 33 | | 57 | 12 | 164 | 122 | 289 | 32 | | 88 | 28 | 633 | 411 | 456 |  |
| 1 | Crct1 | 1.51 | 8.43E-05 | 9.05E-03 | 0 | | 0 | 0 | 59 | 40 | 49 | 0 | | 0 | 0 | 229 | 95 | 98 |  |
| 1 | Cth | -1.67 | 1.13E-03 | 4.78E-02 | 140 | | 145 | 127 | 36 | 57 | 25 | 53 | | 44 | 99 | 11 | 13 | 13 |  |
| 1 | Cux2 | -1.52 | 1.41E-05 | 2.58E-03 | 613 | | 651 | 935 | 297 | 362 | 123 | 550 | | 539 | 677 | 87 | 105 | 79 |  |
| 1 | Cxcl5 | 1.40 | 1.17E-07 | 5.73E-05 | 31 | | 100 | 18 | 360 | 278 | 577 | 85 | | 55 | 124 | 1044 | 885 | 1320 |  |
| 1 | Cytip | 1.01 | 1.63E-05 | 2.88E-03 | 94 | | 152 | 56 | 420 | 316 | 571 | 186 | | 299 | 117 | 1067 | 830 | 763 |  |
| 1 | Entpd3 | 1.71 | 1.30E-08 | 1.01E-05 | 9 | | 14 | 2 | 74 | 36 | 69 | 16 | | 30 | 11 | 185 | 172 | 231 |  |
| 1 | Esrrg | -1.05 | 2.21E-05 | 3.48E-03 | 5424 | | 4731 | 4856 | 1860 | 2381 | 1162 | 4337 | | 4483 | 4470 | 1051 | 852 | 726 |  |
| 1 | Gpr141 | 1.32 | 5.46E-05 | 6.46E-03 | 46 | | 45 | 21 | 167 | 171 | 439 | 84 | | 57 | 30 | 599 | 634 | 729 |  |
| 1 | Havcr2 | 1.06 | 1.38E-06 | 4.07E-04 | 22 | | 31 | 23 | 267 | 197 | 272 | 58 | | 65 | 27 | 560 | 585 | 398 |  |
| 1 | Hlf | -1.14 | 6.65E-04 | 3.43E-02 | 691 | | 630 | 939 | 182 | 318 | 128 | 551 | | 481 | 614 | 116 | 93 | 78 |  |
| 1 | Il18rap | 1.22 | 1.58E-04 | 1.37E-02 | 70 | | 70 | 30 | 224 | 125 | 261 | 64 | | 61 | 53 | 720 | 377 | 338 |  |
| 1 | Il1r2 | 2.50 | 1.16E-14 | 3.07E-11 | 94 | | 117 | 33 | 320 | 176 | 588 | 148 | | 325 | 125 | 2385 | 1808 | 1985 |  |
| 1 | Jaml | 1.23 | 1.43E-05 | 2.60E-03 | 48 | | 21 | 22 | 100 | 92 | 176 | 77 | | 65 | 42 | 245 | 293 | 339 |  |
| 1 | Kcne4 | 1.06 | 3.18E-04 | 2.14E-02 | 66 | | 45 | 30 | 170 | 99 | 183 | 108 | | 110 | 93 | 430 | 257 | 257 |  |
| 1 | Kcnj3 | -1.56 | 1.09E-06 | 3.36E-04 | 2720 | | 2971 | 3558 | 816 | 1363 | 534 | 2112 | | 2711 | 2511 | 435 | 265 | 226 |  |
| 1 | Kcnj5 | -1.23 | 3.22E-06 | 8.07E-04 | 1329 | | 1391 | 1387 | 485 | 637 | 294 | 1064 | | 1075 | 1051 | 203 | 206 | 196 |  |
| 1 | Kcnn2 | -1.28 | 1.88E-04 | 1.56E-02 | 1386 | | 1829 | 3012 | 507 | 1226 | 433 | 2077 | | 1757 | 1669 | 393 | 257 | 245 |  |
| 1 | Klhl33 | -1.14 | 6.11E-04 | 3.32E-02 | 269 | | 360 | 521 | 102 | 128 | 70 | 235 | | 461 | 370 | 41 | 43 | 55 |  |
| 1 | Mogat2 | -1.49 | 7.65E-04 | 3.75E-02 | 282 | | 317 | 324 | 130 | 140 | 40 | 256 | | 312 | 388 | 30 | 43 | 38 |  |
| 1 | Ms4a4a | 1.28 | 9.51E-07 | 3.10E-04 | 201 | | 191 | 117 | 493 | 405 | 761 | 326 | | 458 | 255 | 1800 | 1164 | 1104 |  |
| 1 | Piwil2 | 1.07 | 1.17E-03 | 4.88E-02 | 25 | | 28 | 12 | 73 | 45 | 78 | 32 | | 37 | 30 | 166 | 151 | 91 |  |
| 1 | Ppp2r2c | -2.27 | 9.01E-04 | 4.13E-02 | 138 | | 163 | 202 | 65 | 44 | 9 | 183 | | 212 | 192 | 10 | 8 | 6 |  |
| 1 | Ptpn22 | 1.57 | 1.77E-07 | 7.83E-05 | 57 | | 71 | 46 | 150 | 218 | 359 | 87 | | 93 | 40 | 975 | 617 | 604 |  |
| 1 | Ptx3 | 1.89 | 1.27E-13 | 2.94E-10 | 32 | | 31 | 17 | 247 | 155 | 228 | 27 | | 136 | 250 | 964 | 535 | 850 |  |
| 1 | Rab37 | 1.15 | 1.08E-06 | 3.36E-04 | 76 | | 91 | 36 | 671 | 405 | 719 | 96 | | 116 | 72 | 1646 | 1198 | 1154 |  |
| 1 | Rdh12 | 1.53 | 4.42E-04 | 2.75E-02 | 8 | | 8 | 8 | 25 | 15 | 51 | 10 | | 21 | 11 | 109 | 87 | 72 |  |
| 1 | Rny3 | -2.95 | 1.34E-22 | 1.63E-18 | 991 | | 762 | 1538 | 4297 | 2503 | 5087 | 1228 | | 1046 | 2142 | 303 | 699 | 537 |  |
| 1 | Serpina3h | 1.24 | 7.54E-04 | 3.71E-02 | 29 | | 10 | 13 | 87 | 40 | 38 | 35 | | 63 | 28 | 170 | 98 | 115 |  |
| 1 | Sirpb1b | 1.58 | 4.05E-10 | 4.70E-07 | 132 | | 121 | 32 | 393 | 326 | 688 | 93 | | 114 | 68 | 1398 | 1400 | 1437 |  |
| 1 | Sla | 1.30 | 2.33E-05 | 3.54E-03 | 284 | | 248 | 159 | 553 | 519 | 1363 | 392 | | 422 | 228 | 2081 | 1934 | 2057 |  |
| 1 | Slc38a3 | -1.27 | 3.91E-04 | 2.53E-02 | 1858 | | 1765 | 1466 | 654 | 700 | 201 | 1567 | | 1717 | 1865 | 191 | 200 | 255 |  |
| 1 | Slc7a11 | 1.21 | 2.17E-04 | 1.71E-02 | 45 | | 77 | 33 | 312 | 135 | 415 | 53 | | 63 | 33 | 811 | 514 | 691 |  |
| 1 | Soat2 | 1.01 | 8.13E-04 | 3.90E-02 | 21 | | 31 | 11 | 116 | 56 | 91 | 18 | | 21 | 24 | 219 | 158 | 151 |  |
| 1 | Socs3 | 1.13 | 8.96E-06 | 1.78E-03 | 258 | | 233 | 90 | 739 | 399 | 688 | 338 | | 333 | 599 | 1769 | 1090 | 1169 |  |
| 1 | Sphk1 | 1.65 | 5.49E-07 | 1.96E-04 | 43 | | 42 | 21 | 161 | 75 | 156 | 84 | | 195 | 47 | 604 | 337 | 294 |  |
| 1 | Spint1 | 1.13 | 4.95E-04 | 2.89E-02 | 5 | | 8 | 2 | 37 | 32 | 42 | 2 | | 3 | 0 | 81 | 74 | 92 |  |
| 1 | Tarm1 | 1.45 | 8.83E-06 | 1.78E-03 | 6 | | 8 | 4 | 80 | 34 | 79 | 16 | | 16 | 6 | 205 | 151 | 173 |  |
| 1 | Tg | 1.41 | 5.75E-06 | 1.32E-03 | 330 | | 316 | 203 | 614 | 578 | 1537 | 489 | | 496 | 273 | 2472 | 2310 | 2536 |  |
| 1 | Veph1 | 1.85 | 6.17E-13 | 1.15E-09 | 35 | | 34 | 26 | 258 | 159 | 241 | 27 | | 138 | 255 | 971 | 544 | 871 |  |
| 2 | Adamts17 | -1.01 | 6.56E-04 | 3.41E-02 | 601 | | 481 | 615 | 275 | 351 | 152 | 420 | | 525 | 556 | 123 | 140 | 124 |  |
| 2 | Adrb1 | -1.18 | 1.08E-03 | 4.65E-02 | 495 | | 512 | 596 | 215 | 331 | 108 | 495 | | 488 | 446 | 121 | 86 | 82 |  |
| 2 | Angpt1 | -1.40 | 3.78E-09 | 3.34E-06 | 1804 | | 2757 | 2971 | 1186 | 2016 | 1054 | 1334 | | 1871 | 1414 | 505 | 557 | 555 |  |
| 2 | Asprv1 | 1.60 | 4.27E-04 | 2.69E-02 | 9 | | 12 | 3 | 22 | 11 | 35 | 16 | | 29 | 22 | 85 | 52 | 71 |  |
| 2 | Bcl2l15 | 2.51 | 8.77E-09 | 7.41E-06 | 12 | | 14 | 7 | 25 | 9 | 20 | 18 | | 26 | 13 | 141 | 76 | 84 |  |
| 2 | Cdh20 | -1.71 | 8.32E-04 | 3.95E-02 | 86 | | 73 | 184 | 53 | 73 | 27 | 71 | | 72 | 55 | 19 | 17 | 9 |  |
| 2 | Cxcl1 | 1.55 | 1.58E-07 | 7.18E-05 | 45 | | 62 | 17 | 99 | 66 | 109 | 51 | | 24 | 70 | 370 | 229 | 205 |  |
| 2 | Cxcl3 | 1.63 | 6.34E-04 | 3.38E-02 | 0 | | 7 | 0 | 53 | 17 | 70 | 0 | | 0 | 9 | 236 | 84 | 117 |  |
| 2 | Cxcr2 | 1.23 | 2.17E-06 | 5.82E-04 | 87 | | 99 | 16 | 185 | 131 | 259 | 84 | | 77 | 108 | 459 | 384 | 522 |  |
| 2 | Fbxl5 | 1.04 | 1.53E-04 | 1.35E-02 | 1482 | | 1575 | 1170 | 1925 | 1540 | 3328 | 1214 | | 1646 | 1271 | 5872 | 3419 | 4857 |  |
| 2 | Fign | -1.14 | 2.26E-04 | 1.76E-02 | 1196 | | 1180 | 1491 | 382 | 871 | 394 | 678 | | 750 | 819 | 200 | 253 | 297 |  |
| 2 | Flrt1 | -1.69 | 9.00E-06 | 1.78E-03 | 161 | | 202 | 188 | 91 | 108 | 70 | 117 | | 81 | 147 | 21 | 22 | 43 |  |
| 2 | Fpr1 | 1.56 | 3.22E-04 | 2.16E-02 | 19 | | 21 | 0 | 22 | 28 | 62 | 10 | | 26 | 9 | 121 | 69 | 151 |  |
| 2 | Garem1 | -1.05 | 5.81E-07 | 2.00E-04 | 2597 | | 2767 | 2725 | 1332 | 1372 | 830 | 1457 | | 1608 | 2052 | 515 | 603 | 589 |  |
| 2 | Grb14 | -1.02 | 1.03E-04 | 1.01E-02 | 3253 | | 3706 | 4176 | 1703 | 1912 | 839 | 2730 | | 2959 | 3702 | 826 | 662 | 723 |  |
| 2 | H2-Q10 | 1.30 | 9.78E-04 | 4.37E-02 | 22 | | 30 | 10 | 25 | 24 | 38 | 8 | | 22 | 14 | 68 | 55 | 97 |  |
| 2 | Hdc | 1.34 | 9.27E-08 | 4.92E-05 | 97 | | 104 | 125 | 263 | 139 | 183 | 158 | | 257 | 97 | 606 | 436 | 437 |  |
| 2 | Hp | 1.23 | 1.09E-04 | 1.05E-02 | 99 | | 337 | 42 | 458 | 236 | 368 | 214 | | 235 | 125 | 622 | 577 | 1294 |  |
| 2 | Itgb6 | -1.34 | 4.24E-05 | 5.65E-03 | 909 | | 1271 | 1433 | 510 | 720 | 319 | 577 | | 498 | 759 | 120 | 248 | 247 |  |
| 2 | Kbtbd12 | -1.11 | 2.47E-04 | 1.83E-02 | 2085 | | 2051 | 2113 | 884 | 1036 | 394 | 1617 | | 1514 | 1829 | 285 | 380 | 410 |  |
| 2 | Ky | -2.37 | 3.07E-06 | 7.81E-04 | 389 | | 374 | 737 | 108 | 333 | 71 | 289 | | 351 | 435 | 38 | 43 | 17 |  |
| 2 | Lrrc15 | -1.07 | 1.77E-04 | 1.51E-02 | 297 | | 345 | 351 | 198 | 164 | 114 | 264 | | 268 | 264 | 66 | 87 | 72 |  |
| 2 | Mapt | -1.18 | 9.50E-05 | 9.77E-03 | 1955 | | 1904 | 1736 | 771 | 913 | 333 | 1725 | | 1749 | 1758 | 336 | 265 | 290 |  |
| 2 | Mir703 | 1.57 | 1.03E-03 | 4.51E-02 | 20 | | 18 | 15 | 17 | 19 | 9 | 21 | | 13 | 4 | 54 | 35 | 45 |  |
| 2 | Mirt1 | 1.30 | 2.31E-05 | 3.54E-03 | 25 | | 51 | 20 | 93 | 49 | 120 | 26 | | 24 | 22 | 235 | 216 | 198 |  |
| 2 | Mirt2 | 1.98 | 2.47E-05 | 3.67E-03 | 11 | | 11 | 1 | 25 | 6 | 20 | 6 | | 8 | 13 | 78 | 69 | 52 |  |
| 2 | Mlxipl | -1.64 | 9.74E-10 | 9.04E-07 | 562 | | 573 | 617 | 374 | 297 | 194 | 402 | | 417 | 583 | 88 | 88 | 101 |  |
| 2 | Mmp8 | 1.03 | 3.95E-04 | 2.55E-02 | 82 | | 63 | 8 | 207 | 96 | 182 | 45 | | 69 | 81 | 357 | 251 | 385 |  |
| 2 | Mmp9 | 1.24 | 7.43E-05 | 8.21E-03 | 59 | | 160 | 18 | 393 | 153 | 413 | 82 | | 78 | 97 | 946 | 608 | 731 |  |
| 2 | Mt2 | 1.31 | 4.88E-07 | 1.81E-04 | 879 | | 704 | 384 | 1620 | 729 | 1334 | 359 | | 995 | 891 | 3738 | 2485 | 2962 |  |
| 2 | mt-Tf | -1.14 | 1.05E-03 | 4.57E-02 | 388 | | 254 | 490 | 470 | 163 | 473 | 320 | | 435 | 730 | 141 | 207 | 153 |  |
| 2 | Nr1d1 | -1.04 | 2.71E-04 | 1.95E-02 | 1846 | | 1849 | 2623 | 1218 | 1437 | 570 | 1304 | | 1328 | 1960 | 581 | 563 | 423 |  |
| 2 | Nr3c2 | -1.51 | 2.18E-08 | 1.50E-05 | 2520 | | 3091 | 3592 | 1008 | 1678 | 729 | 1934 | | 1708 | 1776 | 458 | 353 | 397 |  |
| 2 | Nr4a1 | 1.04 | 6.89E-04 | 3.52E-02 | 790 | | 272 | 422 | 396 | 238 | 216 | 2415 | | 2954 | 1068 | 881 | 473 | 400 |  |
| 2 | Prox1 | -1.00 | 1.03E-04 | 1.01E-02 | 1953 | | 1868 | 2135 | 1127 | 1142 | 536 | 1764 | | 2037 | 1914 | 503 | 455 | 443 |  |
| 2 | Rny1 | -2.35 | 6.10E-20 | 3.78E-16 | 1415 | | 735 | 1180 | 1877 | 1800 | 2174 | 1985 | | 1247 | 1074 | 239 | 535 | 379 |  |
| 2 | S100a8 | 1.54 | 2.01E-08 | 1.44E-05 | 327 | | 312 | 36 | 263 | 134 | 281 | 268 | | 178 | 239 | 734 | 506 | 749 |  |
| 2 | Serpina3g | 1.00 | 4.32E-05 | 5.65E-03 | 71 | | 71 | 30 | 176 | 107 | 164 | 69 | | 67 | 41 | 265 | 283 | 353 |  |
| 2 | Siglece | 1.25 | 5.58E-06 | 1.31E-03 | 66 | | 66 | 25 | 114 | 73 | 146 | 71 | | 101 | 85 | 276 | 228 | 300 |  |
| 2 | Slc38a11 | -1.49 | 9.39E-04 | 4.28E-02 | 102 | | 61 | 104 | 40 | 63 | 38 | 92 | | 78 | 85 | 13 | 20 | 17 |  |
| 2 | Stfa2l1 | 2.20 | 2.66E-04 | 1.93E-02 | 7 | | 11 | 0 | 15 | 6 | 15 | 6 | | 8 | 9 | 105 | 33 | 29 |  |
| 2 | Syde2 | -1.01 | 9.13E-05 | 9.53E-03 | 1341 | | 1123 | 1323 | 478 | 723 | 345 | 883 | | 773 | 1082 | 274 | 241 | 257 |  |
| 2 | Thrb | -1.16 | 9.11E-07 | 3.02E-04 | 3747 | | 3601 | 4399 | 1557 | 1846 | 962 | 2743 | | 3433 | 3140 | 563 | 746 | 654 |  |
| 2 | Tnfsf11 | 1.68 | 3.86E-05 | 5.28E-03 | 7 | | 8 | 0 | 34 | 15 | 18 | 0 | | 5 | 0 | 72 | 58 | 81 |  |
| 3 | Rpl15-ps6 | 3.86 | 2.38E-19 | 1.11E-15 | 53 | | 150 | 5 | 15 | 10 | 7 | 101 | | 33 | 50 | 175 | 179 | 97 |  |
| 3 | Fam107a | 1.47 | 5.48E-08 | 3.28E-05 | 539 | | 305 | 276 | 128 | 107 | 104 | 516 | | 993 | 502 | 436 | 277 | 228 |  |

Table S3. RNA-seq DEGs.

Differentially expressed genes (DEGs) determined by RNA-seq analysis of left ventricular tissue. Normalized transcript counts are shown for PKM2^fl/fl^ and PKM2^-/-^ sham and MI hearts. Groups indicated in column 1 are as follows: group 1 = genes regulated by infarction in both PKM2^fl/fl^ and PKM2^-/-^ hearts to differing degrees, group 2 = genes regulated by infarction in only PKM2^-/-^ hearts, and group 3 = genes regulated by infarction in only PKM2^fl/fl^ hearts.
